# Aging, Cellular Senescence, and Glaucoma

**DOI:** 10.14336/AD.2023.0630-1

**Published:** 2024-04-01

**Authors:** Yumeng Zhang, Shouyue Huang, Bing Xie, Yisheng Zhong

**Affiliations:** Department of Ophthalmology, Ruijin Hospital Affiliated Medical School, Shanghai Jiaotong University, Shanghai 200025, China

**Keywords:** glaucoma, cellular senescence, aging, oxidative stress, DNA damage, mitochondrial dysfunction, defective autophagy, epigenetic modifications

## Abstract

Aging is one of the most serious risk factors for glaucoma, and according to age-standardized prevalence, glaucoma is the second leading cause of legal blindness worldwide. Cellular senescence is a hallmark of aging that is defined by a stable exit from the cell cycle in response to cellular damage and stress. The potential mechanisms underlying glaucomatous cellular senescence include oxidative stress, DNA damage, mitochondrial dysfunction, defective autophagy/mitophagy, and epigenetic modifications. These phenotypes interact and generate a sufficiently stable network to maintain the cell senescent state. Senescent trabecular meshwork (TM) cells, retinal ganglion cells (RGCs) and vascular endothelial cells reportedly accumulate with age and stress and may contribute to glaucoma pathologies. Therapies targeting the suppression or elimination of senescent cells have been found to ameliorate RGC death and improve vision in glaucoma models, suggesting the pivotal role of cellular senescence in the pathophysiology of glaucoma. In this review, we explore the biological links between aging and glaucoma, specifically delving into cellular senescence. Moreover, we summarize the current data on cellular senescence in key target cells associated with the development and clinical phenotypes of glaucoma. Finally, we discuss the therapeutic potential of targeting cellular senescence for the management of glaucoma.

## 1. Introduction: aging and glaucoma are intimately linked

Globally, glaucoma causes irreversible blindness (distance visual acuity worse than 3/60) and permanent vision loss as a result of accelerated retinal ganglion cell (RGC) death, axonal degeneration, and optic nerve function damage [1]. This ocular disease encompasses a group of optic neuropathies of which primary open angle glaucoma (POAG) is the most common [2]. Several risk factors, including elevated intraocular pressure (IOP), advanced age, and genetics, have been reported to contribute to the development of glaucoma, among which age has consistently been identified by several epidemiological studies as a major risk factor [3-5]. According to age-standardized prevalence, glaucoma is the second leading cause of legal blindness worldwide and also causes substantial disability before blindness; however, it remains undertreated [6]. Despite this situation, the mechanisms underlying the specific increase in the vulnerability of RGCs to glaucomatous damage as people age remain unclear.

A decrease in the anterior segment outflow facility concomitant with an age-related increase in the prevalence of glaucoma has been reported [7]. The malfunction of the anterior segment outflow tissue, which includes the trabecular meshwork (TM) and Schlemm’s canal, may contribute to the increase in IOP and be associated with most forms of glaucoma [8]. Aging results in a reduction in the number of cells in the outflow system of the normal eye, which is associated with some of the structural changes that occur as we age, such as trabecular thickening and fusion [9, 10]. Compared with age-matched controls, patients with POAG have been found to lose a greater number of TM cells as they age [11, 12]. Moreover, it has been suggested that the absence of TM cells, followed by their replacement with extracellular matrix (ECM) leads to increased resistance to fluid outflow in these patients, thus triggering an increase in IOP [13]. Furthermore, it has been proposed that type VI collagen plays a role in cell-ECM interactions in the TM, with its abnormal accumulation in glaucomatous and aging eyes probably indicating a defect in the trabecular cells [14].

RGCs are retinal neurons that transmit visual information to the brain through long axons [15]. As the function of the aqueous humor outflow apparatus decreases, the number of RGCs in the retina also decreases [16]. In addition, many studies have indicated that the number of RGC axons decreases with aging. Axon loss is estimated at approximately 4500 axons per year in both humans and rhesus monkeys [17]. It appears that the RGCs undergo a period of adaptation and dysfunction prior to irreversible cell apoptosis [18]. However, clinical and rodent studies have demonstrated, however, that older eyes have greater RGC functional deficits when the IOP is elevated acutely [19, 20]. Studies have also demonstrated that the thickness of the nerve fiber layer decreases with age in normal individuals. Furthermore, aging contributes to an abnormal ocular blood flow, blood vessels narrowing, and endothelial dysfunction [21, 22]. Moreover, a close association between these features and the development of glaucoma has been reported [23]. In light of these findings, it is believed that glaucoma is pathophysiologically related to aging.

It must be recognized that aging alone does not lead to diseases. There is a greater likelihood that aging, as a result of a series of changes, leads to a more fragile vascular system, connective tissue, and RGCs, which means greater vulnerability to insults and higher risk for glaucoma development over time. Moreover, the speed and extent of the insults are modulated by genes and environmental factors that determine an individual’s aging process [24]. Despite the limited understanding of the aging process and its biological mechanisms, studies conducted over the last few decades have identified common molecular and cellular hallmarks associated with aging, including genomic instability, telomere attrition, epigenetic changes, proteostasis loss, deregulated nutrient sensing, mitochondrial dysfunction, cellular senescence, stem cell exhaustion, and altered intercellular communication [25]. The identification of hallmarks of aging facilitates the conceptualization of aging research and raises the prospect of impeding various age-related diseases by targeting the aging process. Among these hallmarks, cellular senescence has attracted considerable attentions since it is not only directly implicated as a major direct driver of aging and age-related diseases, but also as a potential druggable mechanism involved in the prevention or treatment of multiple aging comorbidities. However, the role of cellular senescence plays in the pathogenesis and treatment of glaucoma hasn’t been adequately explored.

In this review, we delve into cellular senescence, which is a homeostatic biological process that plays a key role in driving aging and age-related diseases, to figure out its biological mechanisms underlying glaucoma pathogenesis and potential as a promising target for glaucoma treatment [26].

## 2. Cellular senescence

**Figure 1.**
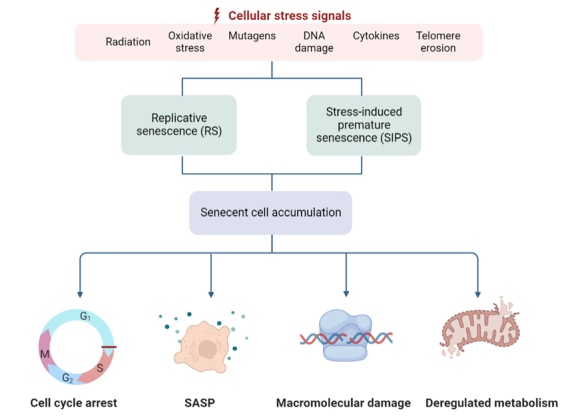
**Causes and consequences of cellular senescence**. Cellular senescence is a complex process triggered by a myriad of pro-senescent stimuli related to ageing, such as radiation, oxidative stress, mutagens, DNA damage, cytokines, telomere erosion, among others. SIPS and RS are the two main forms of cellular senescence and result in accumulation of senescent cells. This occurs in ageing and neurodegeneration and leads to cell cycle arrest, SASP, macromolecular damage, and deregulated metabolism, which may ultimately contribute to organ and tissue decline. SASP, senescence-associated secretory phenotype; RS, replicative senescence; SIPS, stress-induced premature senescence.

As reported previously, fibroblasts have a limited replication capacity, suggesting that aging also occurs at the cellular level [27]. This phenomenon is currently known as cellular senescence and represents a state of irreversible growth arrest and resistance to cell apoptosis in response to cellular damage and stress. Senescent cells become enlarged with a flattened morphology and lose their proliferative capacity irreversibly. In several age-related diseases, including glaucoma, the accumulation of senescent cells causes aging and loss of tissue function [28]. Cellular senescence can be classified into several types according to the initial senescence-inducing signals; i.e., replicative senescence (RS), stress-induced premature senescence (SIPS), oncogene-induced senescence, therapy-induced senescence, secondary senescence and post-mitotic cell senescence [29]. Cellular senescence is characterized by multiple interconnected hallmarks, including cell cycle arrest, senescence-associated secretory phenotype (SASP), macromolecular damage, and deregulated metabolism, regardless of the type of senescence [30] (Fig. 1). SIPS and RS are the two main forms of cellular senescence, which refer to the irreversible cessation of cell growth and division, and both are dependent on two major pathways. In the first pathway, p53 and p21^WAF-1^ are activated by the occurrence of DNA damage, or telomere damage/ shortening. In turn, the second pathway involves the accumulation of p16^INK4a^, via mitogen-activated protein kinase (MAPK) signaling as an intermediate step [31]. However, these two types of cellular senescence differ in their underlying causes and mechanisms. RS is induced by telomere erosion and DNA damage responses (DDRs), whereas SIPS can be elicited by other stressors, including but not limited to oxidative stress, radiation, mutagens, cytokines, epigenetic changes and genomic instability [26, 32]. Although RS was traditionally thought to be dependent on telomere shortening, and SIPS was believed to be a telomere-length-independent process, recent research indicates that there is sometimes a blurry line between these two types of senescence [30, 33]. In addition to shortening telomeres and triggering RS prematurely, stress factors may also cause telomere uncapping without shortening, thereby leading to telomere-dependent SIPS [33]. Accordingly, it has been suggested that all forms of senescence are caused by cellular stress responses, which has now become generally accepted. Despite the fact that the exact types of cellular senescence that contribute to the disease process in glaucoma remain unknown, it has been suggested that the accumulation of senescent cells in glaucoma with age may mainly result from exposure to stress factors, rather than RS [7, 34].

### 2.1 Evidence of cellular senescence in aging

As mentioned earlier, cellular senescence is a key manifestation of aging at the cellular level and defined as a cellular response to a wide variety of stresses. In aging tissues, the accumulation of senescent cells is irreversible, as cellular damage accumulates over time, and is accompanied by a decline in immune function and intracellular homeostasis [35]. Various tissues of elderly individuals as well as individuals with premature aging syndromes have been reported to contain senescent cells [36-38]. In fact, several studies have indicated that cellular senescence can be caused or accompanied by multiple processes, including DNA damage, oncogenic mutations, production of reactive metabolites, and proteotoxic stress, that are also associated with aging [39-41].

Recent studies have been shown that the removal of senescent cells may delay the onset of age-related diseases [37, 42-44]. Inducible elimination of p16^INK4a^-positive senescent cells was achieved using a novel transgene, INK-ATTAC, after drug administration [42]. In addition, the selective elimination of senescent cells from mice aged ≥1 year extended the life expectancy of these animals and delayed age-related cellular and tissue dysfunction [37]. Moreover, the removal of p16^INK4a^-positive cells also reduced the deterioration of several organs, including the kidneys, heart, and fat, as a result of aging [37]. Using a similar line of investigation, another recent study demonstrated that the clearance of p16^INK4a^-positive senescent astrocytes and microglia halted neurodegeneration and preserved cognitive function by reducing the accumulation of aberrantly hyper-phosphorylated of tau protein [43]. Thus, cellular senescence may be crucial in the process of neurodegenerative diseases associated with aging.

### 2.2 Mechanisms of cellular senescence

The retina is comprised largely of highly specialized post-mitotic cells, including retinal pigment epithelial cells, photoreceptors and RGCs, which have high metabolic activity and oxygen consumption [45]. Thus, it is susceptible to oxidative stress, cellular hypoxia and other concomitant aging processes including DNA damage, mitochondrial dysfunction, defective autophagy, epigenetic factors, and chronic inflammation [46-48]. These phenotypes either partially or completely participate and interplay in senescence, forming positive feedback loops that trigger, develop, and maintain the senescent cell state, thus may predispose to glaucomatous injury. In this section, the potential mechanisms of glaucomatous cellular senescence are mainly discussed.

#### 2.2.1 Oxidative stress

It has been established that oxidative stress is a key factor in accelerated aging, as the formation of reactive oxygen species (ROS) during normal oxygen metabolism causes oxidative damage to the cells, which accelerates the process of cellular senescence and ultimately quickens the aging process [49, 50]. Increased ROS may be a result of the reduced activity of Nuclear Factor erythroid 2-Related Factor 2, an antioxidant regulating transcription factor [51]. Different pathways involved in cellular senescence are driven by ROS, such as the activation of MAPK signaling pathway, PI3K-AKT-mTOR pathway, and the transforming growth factor-β (TGF-β) pathway, all of which result in p15, p21 and p27 upregulation [52-54].

It is believed that a reduction in oxygen supply and irregularities in vascular perfusion contribute to oxidative damage in glaucoma [55]. Patients with glaucoma exhibit elevated levels of oxidative stress markers, particularly malondialdehyde, in the serum and aqueous humor. In addition, some anti-oxidative stress markers were found to be elevated in aqueous humor, indicating that the eye may respond to oxidative stress in a protective manner [56]. Another recent study demonstrated that a sudden and marked increase in IOP in glaucoma led to an increase in the levels of oxidative stress markers and premature senescence markers in the anterior segment; moreover, the levels of oxidative stress markers were positively correlated with the peak preoperative IOP and age [57]. The gradual accumulation of evidence suggests that oxidative stress is an important contributor to the damage to the TM and RGCs in glaucoma [56, 58, 59]. A high level of the metabolic products of lipid peroxidation and DNA adducts has been reported in the TM, which can activate senescence markers such as p16^INK4a^ and p21, resulting in the accumulation of senescent cells [60, 61]. The reduction of the expression and activity of antioxidant proteins, such as peroxidase 6 (Prdx6), resulted in the accumulation of ROS and pathological changes in aging or glaucomatous TM cells, whereas the overexpression of Prdx6 reduced the accumulation of senescent cells by inhibiting oxidative stress [61]. In addition, researchers have proposed that an intracellular ROS superoxide burst could be responsible for triggering RGC death following axonal injury [62, 63]. Intraocular oxidative stress can damage the RGC directly or indirectly by triggering caspase activation [64]. Surprisingly, senescent cells in the optic nerve and TM can disrupt the tissue microenvironment by producing ROS [34]. By increasing ROS production, senescent cells may cause adjacent nonsenescent cells to malfunction, which may lead to the development of glaucoma [65, 66]. These findings suggest the interesting possibility that oxidative stress is responsible for both the onset and progression of glaucoma.

#### 2.2.2 DNA damage and DDR activation

A coordinated series of events known as DDR occur in response to DNA damage. Histone H2AX phosphorylated on Ser139 (γH2A.X), which is a signal that facilitates the focal assembly of checkpoint and DNA repair factors after DNA damage, is an indicator of DDR [67]. Persistent DDR is one of the main characteristics of senescent cells [68, 69]. When DNA damage occurs, the kinase cascade is activated, initially involving the serine/threonine-nonspecific kinases ATM and ATR [70], followed by the checkpoint serine/threonine kinases CHK1 and CHK2, which ultimately lead to the activation of the p53/p21 signaling pathway [71, 72]. The production of ROS, either directly from hydrogen peroxide exposure or indirectly via endogenous production (i.e., mitochondrial dysfunction, as detailed in subsection 2.2.3), can cause DNA damage, the activation of DDR and cell senescence [73, 74]. In addition, DDR may also play a role in initiating the development of SASP via the activation of the nuclear factor-kappa B (NF-κB) transcription factor pathway, which is the most characteristic functional feature of cellular senescence [75, 76].

Increasing evidence indicates that DNA damage may provide a pathway that links the risk factors of glaucoma and RGC death. As evidenced by both RNA sequencing and γ-H2A.X immunostaining in the DBA/2J (D2) mouse model (a classic model of chronic inherited glaucoma), ROS and DNA damage were increased within RGCs early in the disease process [77]. Moreover, 8-hydroxy-2'-deoxyguanosine (8-OH-dG) and 8-hydro-xyguanosine (8-OHG) are biomarkers of oxidative damage in DNA and RNA, respectively [78]. Previous studies using human samples showed that 8-OH-dG was increased in the serum, aqueous humor, and TM specimens from patients with glaucoma compared with controls [79, 80]. Similarly, in a laser-induced chronic glaucoma model in rhesus monkeys, 8-OHG and γH2A.X levels were found to be much higher than those in their control counterparts in different regions of neurons, which ultimately led to neuronal apoptosis and autophagy activation [81]. In turn, 8-OH-dG and 8-OHG were also upregulated in RGCs, suggesting that an elevated IOP increases the level of oxidatively damaged DNA/RNA, which may contribute to the progression of glaucoma [82]. However, further research is needed to determine whether DDR activation in glaucoma contributes to glaucomatous RGC degeneration by causing cellular senescence.

#### 2.2.3 Mitochondrial dysfunction

An array of cellular functions is carried out by mitochondria, including (1) regulating intracellular calcium levels, which affect intracellular signaling, neuronal excitability, and synaptic transmission as well as (2) maintaining cellular homeostasis and metabolic functions, including oxidative energy metabolism [83]. Many pathological conditions associated with aging, including cancer, neurodegenerative diseases, and metabolic disorders, are associated with impaired mitochondrial function and morphology [84]. Many features of the senescent phenotype, especially the secretion of SASP, can be reversed by mitochondrial ablation [85]. The DDR can initiate mitochondrial dysfunction via the p38 MAPK and TGF-β pathways or by SASP via the upregulation of NF-κB. Because of the elevated levels of ROS caused by mitochondrial dysfunction, additional DNA damage and SASP secretion were triggered, which ultimately resulted in the establishment of positive feedback loops that stabilize senescence [86, 87]. To generate membrane potentials, neurons require an abundance of adenosine triphosphate (ATP) to regulate ion gradients across membranes. Mitochondrial dysfunction develops with age and is associated with the degeneration of all neuronal cell types. Of note, mitochondria are primarily concentrated in the unmyelinated regions of mammalian RGC axons, where many mitochondria are essential for the transmission of information to the brain [88]. An analysis of the metabolomics profiling of POAG indicated that the presence of mitochondrial dysfunction and senescence-like alteration was strongly associated with glaucoma [89]. Furthermore, because there was no significant difference in age between patients with POAG and controls, it is tempting to speculate that premature mitochondrial dysfunction contributes to POAG risk by increasing the aging phenotype. A further independent study of the involvement of mitochondria in glaucoma has been conducted in D2 mice, which revealed that, with age, the retinal levels of nicotinamide adenine dinucleotide (NAD^+^, a key molecule in energy and redox metabolism) decreased, thus rendering neurons more susceptible to disease-related insults [77]. This finding also illustrated the contention that mitochondrial dysfunction may precede the onset of neurodegeneration in RGCs.

In addition, mitochondrial DNA (mtDNA) deletions or mutations have been found to be associated with aging and age-related diseases [90]. The accumulation of somatic mtDNA mutations that occur with aging leads to a loss of mitochondrial function. The resultant decline in energy capacity, increase in oxidative damage, and increase in apoptosis lead to cellular loss, resulting in organ failure [91]. However, it remains unclear how mtDNA mutations accumulate and how they are relevant to aging, as mtDNA mutations are present at relatively low overall levels in normal aging tissues. Whether genetic variation in mitochondria plays a role in POAG has been investigated by conducting an association analysis of mitochondrial SNPs and haplogroups in 721 patients with POAG and 1951 healthy individuals [92]. MT-ND4 and MT-CYB mutations in the mtDNA were found to alter mitochondrial respiratory chain function and lead to changes in cellular energy metabolism, which may play a role in optic neuropathy or glaucoma [92]. Mouse models that accumulate high levels of mtDNA mutations because of impairments in the proofreading function of mitochondrial polymerase c (PolG) reportedly develop phenotypes consistent with accelerated aging [93, 94]. The PolG transgenic mice exhibited accelerated-age-related loss of retinal function, as measured by dark-adapted electroretinogram, as well as increased neuronal vulnerability to external stresses, such as an acutely elevated IOP [94]. However, further experimental evidence is still required to fully elucidate the role of accumulated mtDNA mutations in the promotion of cellular senescence in glaucoma.

#### 2.2.4 Defective autophagy/mitophagy

As a physiological necessity, autophagy normally maintains cell survival, development, and intracellular homeostasis by degrading abnormal substances, including misfolded or aggregated proteins, damaged mitochondria, excess or damaged lipids, and other cellular debris and malfunctioning organelles, especially in situations of starvation or other stresses [95]. Autophagy appears to become defective in autophagy with aging, in association with changes in the activation status of the mechanistic target of rapamycin (mTOR) and adenosine 5'-monophosphate-activated protein kinase pathways [96, 97]. Dysregulation of autophagy has been implicated in a wide range of diseases, including glaucoma [98, 99]. Recent evidence has shown that the autophagic process becomes dysfunctional during the progression of glaucoma, regardless of the presence of many autophagy-stimulating factors (e.g., ROS, oxidized lipids, and cytokines) present [3, 98]. Senescence-associated beta-galactosidase (SA-β-gal), which indicated an abnormal activity of the lysosomal enzyme β-galactosidase at pH 6.0, is a frequently used marker of senescence [33]. Glaucomatous TM cells showed increased SA-β-gal staining and cellular lipofuscin, along with a reduced LC3-II/LC3-I ratio [100]. Autophagy was not activated after exposure of glaucomatous cells to hyperoxia, which indicates a dysregulated autophagic capacity. Another recent study indicated defective autophagy in TM cells in D2 mice, characterized by increased levels of p62/ SQSTM-1 (an autophagy receptor) [101]. It is thought that p62 serves as a marker of autophagy flux, as it normally interacts with LC3-II for degradation, but accumulates when autophagy is inhibited [102]. Furthermore, after exposure to glaucoma-related stimuli, high levels of mTOR activity and p62 in RGCs, along with low levels of the autophagy-related proteins ATG12-ATG5 and ATG4 and of the BECN1/Beclin1 and LC3-II/LC3-I ratios, may lead to the impairment of the autophagic flux, thereby accelerating the loss of RGCs [103]. Defective autophagy can cause damage to the outflow path of the TM, thus promoting the death of RGCs, which in turn contributes to the deterioration of glaucoma.

Furthermore, defective autophagy is associated with a shift in the cell phenotype to senescent cells, which contributes to a loss of tissue homeostasis. In several organisms, the activation of autophagy has been shown to increase longevity and exert geroprotective effects [104]. It has been demonstrated in various animal models that rapamycin, which is an mTOR inhibitor that activates autophagy, extends the lifespan and reduces markers of senescence and SASPs [105]. A previous study showed that pharmacological inhibition of autophagy reduced upregulation of SA-β-gal, indicating that the occurrence of SA-β-gal is mediated by autophagy [106]. In light of these findings, the modulation of autophagy has been proposed as a targeted therapeutic approach for preventing aging or treating aging-related diseases, including glaucoma. However, future studies should attempt to explore the mechanisms linking defective autophagy to cellular senescence in glaucoma.

Not only soluble cell fractions such as proteins are degraded via autophagy, but also organelles, such as mitochondria, during selective autophagy [107, 108]. GATA4 is a zinc-finger transcription factor that links downstream NF-κB activation with upstream DNA damage, thereby forming a pathway for regulating cellular senescence [109]. GATA4 is normally degraded by selective autophagy mediated by p62, while in conditions of defective autophagy, accumulated GATA4 is responsible for activating the NF-κB pathway and thus contributing to SASP regulation [109]. However, there is a need for further exploration of whether GATA4 and selective autophagy play a vital role in the pathogenesis of glaucoma. PTEN-induced kinase 1 (PINK1)/Parkin RBR E3 ubiquitin-protein ligase (PARKIN) signaling-induced mitophagy is one of the best-described forms of selective autophagy, partly because mitophagy dysfunction is linked to various pathologies, including neurodegenerative diseases and age-related ocular diseases [110]. Mitophagy is reduced in aged cells, which leads to dysfunctional mitochondrial accumulation and ROS-induced senescence [111]. In fact, PINK1-PARKIN-mediated mitophagy plays a crucial regulatory role in attenuating cellular senescence in several diseases [112-114]. Although strong findings of reduced mitophagy were not obtained in D2 mice, several recent studies have demonstrated the protective effect of promoting PINK1/PARKIN-mediated mitophagy in RGCs in models of glaucoma (Table 1) [115-117]. Optineurin (OPTN) is a receptor that facilitates the PARKIN-mediated mitophagy pathway, with mutations in OPTN resulting in POAG [118, 119]. Transgenic mice expressing E50K-OPTN were reported to have elevated levels of LC3-II and loss of RGCs with aging [120]. Furthermore, analyses revealed that E50K-OPTN mice exhibited dysfunction of the autophagy-lysosome pathway, decreased number of mitochondria, and increased formation of autophagosomes, implying that mitophagy is responsible for the death of RGCs (Table 1) [120, 121]. Interestingly, another glaucoma-associated variant of OPTN, M98K, also triggered autophagy-dependent retinal cell death [122]. However, a recent study indicated that mutations in OPTN may cause glaucoma via mechanisms other than defective mitophagy [123]. Therefore, the identification of novel treatment options for glaucoma may depend on understanding how mitophagy functions in glaucoma under different conditions.

**Table 1:** Effects of promoting or inhibiting mitophagy on RGC in models of glaucoma.

Organism	Model	Assay Conditions	Effects in glaucoma	Reference
**Mouse**	Microbead model that elevates IOP	Mitochondrial UCP2 knock-out	↑Mitophagy ↓Oxidative stress ↓RGC loss	[115]
**Rat**	Chronic model induced by translimbal laser photocoagulation of the TM	Overexpression of OPA1	↓RGC cytotoxicity and apoptosis ↑Mitochondria fusion and mitophagy ↑RGC survival	[116]
**Rat**	Chronic model induced by translimbal laser photocoagulation of the TM	Overexpression of PARKIN	↓RGC loss ↓GFAP expression ↑Optineurin expression ↑Mitochondrial health and partially restored dysfunction of mitophagy	[117]
**Mouse**	OPTN E50K mutant mouse model	OPTN E50K knock in	↑the Bax pathway and oxidative stress ↓Mitochondria ↑Autophagosome formation ↑Autophagy-lysosome pathway dysfunction	[120, 121]

UCP2, Uncoupling Protein 2; OPA1, Optic Atrophy Type 1; PARKIN, Parkin RBR E3 ubiquitin-protein ligase; IOP, intraocular pressure; RGC, Retinal ganglion cell; GFAP, Glial fibrillary acidic protein; TM, Trabecular meshwork; OPTN, Optineurin.

#### 2.2.5 Epigenetic modifications

Epigenetic reprogramming and chromatin reorganization occur during senescence, accompanied by a decline in organismal functions [124]. DNA methylation (i.e., the presence of methyl groups at CpG dinucleotides) is the best-studied marker of epigenetic reprogramming in senescence; furthermore, altered DNA methylation (generally low levels) can cause various diseases of aging, including cancer and degenerative diseases [125, 126]. Moreover, DNA methylation promotes mitochondrial dysfunction and SASP production, in turn stabilizing other senescence building blocks [127, 128]. A recent study has established the first direct connection between a defective epigenetic regulatory machinery and genetic forms of optic nerve degeneration [129]. It reported a splicing mutation in the methyltransferase-like 23 (METTL23) gene, which encodes a histone arginine methyltransferase, in a Japanese family spanning three generations of patients with normal tension glaucoma. METTL23 deficiency suppresses H3R17 dimethylation and ultimately triggers NF-κB-mediated inflammation; thus, it can be speculated that METTL23 mutation may lead to SASP production and the subsequent aging phenotypes. A greater effort should be made to establish more cogent evidence to prove that DNA methylation directly contributes to the pathologies of cellular senescence.

Furthermore, the extensive and expanding realm of non-coding RNAs (ncRNAs), such as long non-coding RNAs (lncRNAs), microRNAs (miRNAs), and circular RNAs, has emerged as influential epigenetic factors capable of exerting an impact on the aging process [130]. However, the majority of studies have predominantly concentrated on miRNAs. Through their interaction with the 3' untranslated region of mRNA molecules, miRNAs play a crucial role in regulating the translation of specific genes that typically promote cellular livelihood, thus become a central focus of research concerning cellular senescence [131, 132]. Despite the limitation of research in this area, an increasing body of evidence in recent years has shed light on the link between ncRNAs and gene expression related to the senescent phenotype (as detailed in section 3) [133, 134].

## 3. Roles of cellular senescence related molecules in glaucoma

**Figure 2.**
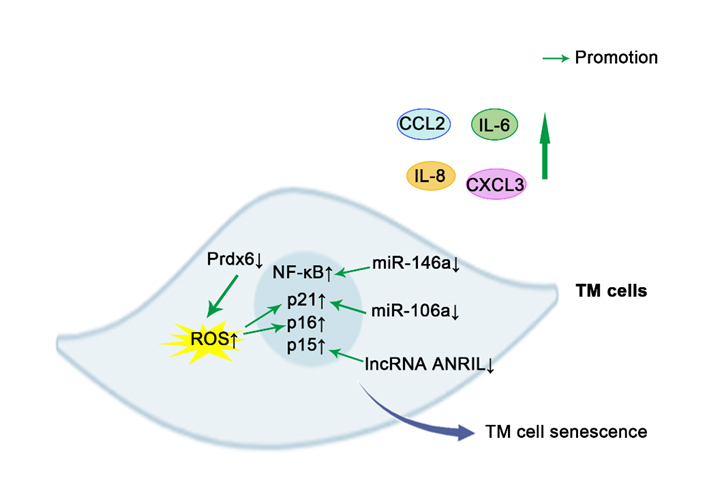
**Schematic representation of pro-senescence mechanisms in TM cells**. The prevention of Prdx6 increases the levels of ROS, thus leading to the overstimulation of p21, p16, and resultant deleterious effects. Downregulation of miR-146a and miR-106a promotes some characteristics of aging cells in TM cells by upregulating NF-κB and p21, respectively. lncRNA ANRIL deficiency significantly upregulated the expression of p15, thus yielding senescence phenotypes in steroid-induced glaucoma. SASPs, including IL-6, IL-8, CCL2, CXCL3, are overproduced in senescence TM cells. CCL2, chemokine (C-C motif) ligand 2; CXCL3, Chemokine (C-X-C Motif) Ligand 3; TM cell, Trabecular meshwork cells; Prdx6, peroxidase (Prdx) 6; p16, p16^INK4a^; Interleukin-6; IL-8, Interleukin-8; NF-κB, transcription factors nuclear factor-kappa B; ROS, reactive oxygen species; p21, p21^WAF-1^; p15, p15^INK4b^; Green arrows indicate promotion. ↑indicates increase, and ↓ indicates reduction.

**Figure 3.**
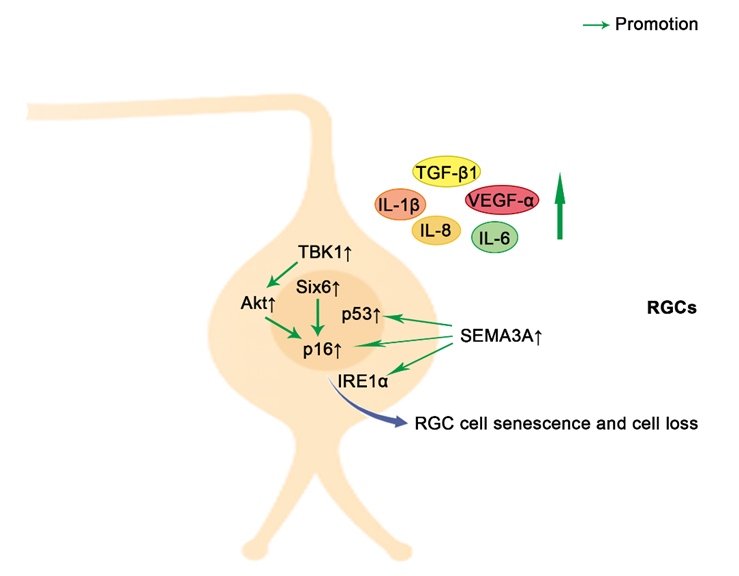
**Schematic representation of pro-senescence mechanisms in RGCs**. The upregulation of Six6 leads to an increase in p16 expression with a subsequent cellular senescence. Elevated IOP increases TBK1 expression, leading to RGC senescence and death through the upregulation of p16 via Akt. The secreted SEMA3A induces a marked increase of p53, p16, and IRE1α, thus contributing to paracrine propagation of senescence. SASPs, such as IL-6, IL-1β, IL-8, TGF-β1 and VEGF-α, are elevated when retinal damage is caused by IOP elevation or retinal ischemia. Akt, V-akt murine thymoma viral oncogene homolog; SEMA3A, semaphorin3A; IRE1α, inositol-requiring enzyme 1α; VEGF-α, vascular endothelial growth factor-α; TGF-β1, Transforming growth factor (TGF)-beta1;RGCs, Retinal ganglion cells; TBK1, TANK binding kinase 1; Six6, Sine oculis-related homeobox 6; p16, p16^INK4a^; IL-1β, Interleukin-1β; IL-6, Interleukin-6; IL-8, Interleukin-8; Green arrows indicate promotion. ↑indicates increase, and ↓ indicates reduction.

**Figure 4.**
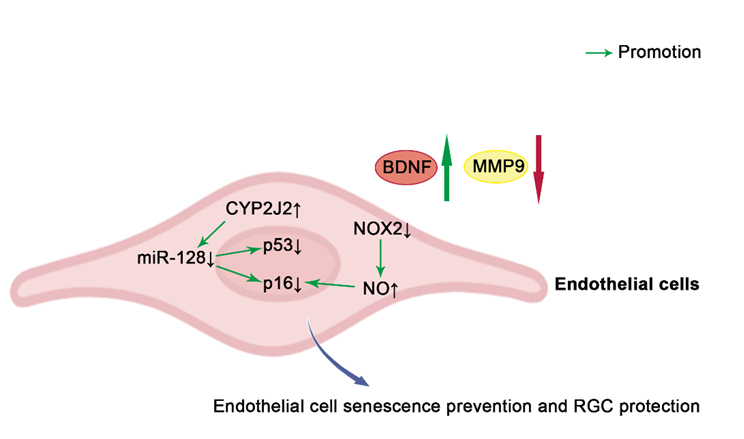
**Schematic representation of anti-senescence mechanisms in endothelial cells**. The overexpression of CYP2J2 inhibits vascular endothelial cell senescence by reducing the expression of p53 and p16 and restoring BDNF and MMP9 levels via miR-128. Blockade of NOX2 or arginase may inhibit premature senescence by inhibiting p16 expression and reducing SASP release through an NO dependent pathway. p16, p16^INK4a^; NOX2, NADPH oxidase 2; NO, nitric oxide; BDNF, brain-derived neurotrophic factor; MMP9, Matrix metallopeptidase 9; CYP2J2, cytochrome P450 2J2; Green arrows and red arrows indicate promotion and inhibition, respectively. ↑indicates increase, and ↓ indicates reduction.

Increasing evidence suggests the close connection between pathways involved in cellular senescence with glaucoma. The major risk factors for the development of glaucoma are high IOP and age, both of which are associated with an increased burden of senescent cells [1, 135]. As a general rule, senescent cells are primarily responsible for causing degenerative changes through secreted SASP, which in turn promotes the maintenance and diffusion of senescence via autocrine and paracrine mechanisms [39, 136]. This section discusses the roles of several representative cells, including TM cells (Fig. 2), RGCs (Fig. 3), and vascular endothelial cells (Fig. 4), in the maintenance of intraocular homeostasis as well as the roles of cellular senescence-related molecules in these cells in glaucoma. Furthermore, it summarizes the current knowledge regarding cellular senescence in key target cells involved in the development and clinical characteristics of glaucoma.

### 3.1 Senescent Cells and Degenerative Phenotypes

Senescent cells have been implicated in numerous age-associated degenerative phenotypes, encompassing both normal and pathological conditions. Extensive evidence suggests that senescent cells play a pivotal role in driving degenerative changes, primarily through the secretion of proteins, collectively referred to as the SASPs. In the mouse model of ocular hypertension and in human glaucomatous retinas, researchers observed an elevation in the presence of senescent cells [70]. Aged retinas exhibit a heightened susceptibility to respond more vigorously to stress associated with IOP compared to young tissues [137]. Consequently, they are more prone to sustaining damage in forms of inflammation and senescence, which entails the secretion of SASPs and destabilization of the extracellular matrix. Moreover, repeated mild stresses exerted on young retinas have been found to accelerate DNA methylation age, indicating a direct influence of repetitive stress on retinal senescence [137]. IL-6, IL-1β, IL-8, CCL2, and other specific components of the SASP have been identified as influential factors that reinforce senescence [46]. Their release has been observed in human glaucomatous retinas or animal models of glaucoma, thereby contributing to chronic inflammation and subsequently leading to the death of RGCs and, ultimately, vision loss [22, 70, 138, 139].

Furthermore, senescent cells can elicit a senescence response through autocrine reinforcement or paracrine transmission, which may elucidate certain deleterious effects associated with the aberrant accumulation of senescent cells during aging [140]. In the retinal ischemia model, RGCs exhibited the initial expression of senescence markers following stress, with senescence subsequently propagating from neurons to retinal microglial cells and the vasculature through bystander signals [22]. Moreover, upon 30 mmHg IOP elevation, various cell types in addition to RGCs demonstrated expressions of several senescence-associated hallmarks, indicating the paracrine nature of senescence [137].

### 3.2 TM cells

Patients with glaucoma exhibit a significant increase in senescent cells in the outflow pathway, which may lead to resistance to atrial outflow, and consequently, an increase in IOP. The intensity of SA-β-gal staining in tissues from glaucoma donors was found to be significantly increased, particularly in the cornea and outflow pathway [34]. In *vitro*, a biochemical analysis of aged and glaucomatous TM cells has revealed increased levels of the senescence markers, p16 and p21, with increased SA-β-gal activity, accompanied by a reduction in telomerase activity [61]. The expression of miRNAs is related to the induction of RS and SIPS in TM cells. An increase in miR-146a expression in senescent TM cells may serve as a preventative measure against the excessive production of inflammatory mediators, thus limiting some of the potentially deleterious effects of the SASP on the physiology of TM cells [138]. Another study reported that the downregulation of miR-106a may promote some of the characteristics of aging cells by upregulating p21 in oxidative stress-induced SIPS in TM cells [133]. The proteasome alleviates the effects of oxidative stress by eliminating the misfolded proteins generated via both direct damage to DNA caused by ROS and oxidative covalent modification of previously synthesized proteins [141, 142]. Chronic oxidative stress results in a significant decrease in proteasome activity, which is associated with an increase in senescent cells among TM cells [143]. It has been noted that proteasome expression decreases with aging in other tissues, which could be attributed to the appearance of senescent cells [144, 145]. Moreover, as mentioned above, Prdx6 limited the levels of ROS, thus preventing overstimulation of p21, p16, and resultant deleterious effects [61]. These results indicate that oxidative stress-induced cellular senescence may impair the intracellular proteasome system and the ability to regulate outflow resistance, leading to malfunction of the outflow pathway. Furthermore, a recent study has found that senescence leads to the stiffening of TM cells, which is accompanied by upregulation of vimentin, F-actin, and Wnt antagonists, such as secreted frizzled related protein-1 (SFRP1) [146]. It is likely that senescence and the resulting SFRP1 expression trigger further senescence and stiffening in neighboring cells, which would promote and spread the senescent phenotype and potentially lead to an increase in IOP and glaucoma progression. A previous study showed that, in steroid-induced glaucoma, cellular senescence exhibited the top enrichment scores in pathway analyses and was the key factor in steroid-induced IOP elevation [134]. The long noncoding RNA ANRIL is involved in the regulation of senescence through the repression of its neighboring gene p15 [147, 148]. ANRIL deficiency in cultured TM cells and mouse models yields similar senescence phenotypes, whereas p15 suppression helps TM cells combat steroid-induced lesions. These findings suggest that cellular senescence may play a significant role in mainstream types of glaucoma.

### 3.3 RGCs

A recent bioinformatics meta-analysis of POAG-related genes concluded that cellular senescence and inflammation plays a key role in RGC degeneration in glaucoma [149]. Patients with glaucoma exhibited increased senescence, as indicated by a marked increase in the level of SA-β-gal in the ganglion cell layer (GCL) [70]. Recent studies have suggested that aged retinas are more sensitive to mild IOP elevation and that old RGCs express significant numbers of senescence markers upon IOP-associated stress, including uPAR, p16^INK4a^ and p19^ARF^ [137]. Interestingly, repeated stress accelerated the appearance of senescence features in young retinas, accompanied by elevated levels of SASP and increased DNA methylation age. These results indicated that RGCs exhibit characteristics of cellular senescence with a high IOP. Another study also revealed that increased IOP was associated with the upregulation of p16^INK4a^ in the acute IOP elevation mouse model, whereas p16KO mice were protected from elevated IOP-induced cell death [70]. Early clearance of p16^INK4a^-positive cells with ganciclovir protected unaffected RGCs from senescence and apoptosis, resulting in a strong protective effect on RGC survival and visual function [150]. Moreover, a risk variant of SIX6 (His141Asn) has been shown to be associated with an increased susceptibility to POAG via increases in p16^INK4a^ transcription and RGC senescence [70]. Using a similar line of investigation, another study demonstrated that an elevated IOP increased TANK-binding protein 1 expression, leading to RGC senescence and death through the upregulation of p16^INK4a^ binding [151]. Further research revealed an increased expression of secretory molecules such as interleukin-1β (IL-1β), which are part of SASP, in the presence of retinal damage caused by IOP elevation [70, 139]. Retinal ischemia triggers cellular senescence via the upregulation of classical senescence-associated proteins, such as p53, p16^INK4a^, and γH2A.X, and multiple SASP marker genes, including matrix-degrading enzyme plasminogen activator inhibitor 1 (*Pai1*), *TGF-β1*, *IL-6*, *IL-1β*, and vascular endothelial growth factor-α (*VEGF-α*) [22]. Furthermore, the senescent state of RGCs was dependent on the expression of semaphorin3A (SEMA3A); in turn, the suppression of SEMA3A blocked the paracrine propagation of senescence [22]. In light of these findings, it appears that RGC senescence is a phase that precedes RGC death, and that inhibition of RGC senescence may be beneficial for rescuing RGCs and improving visual function.

### 3.4 Vascular endothelial cells

The retinal vasculature plays a crucial role in the development of acute and chronic glaucoma. Multiple independent studies have reported a reduced retinal blood flow and capillary density in patients with glaucoma [152, 153]. In patients with glaucoma with impaired retinal vascular function and reduced retinal vascular reactivity, RGCs and their axons are exposed to increased oxidative stress, which may lead to further progression of glaucomatous damage [154]. Moreover, it is becoming increasingly evident that cellular senescence leads to impaired angiogenesis [22, 155]. A reduction in vascular density and an increase in permeability occur as a result of the cellular senescence of endothelial cells, both of which are associated with the development of age-related neurodegenerative diseases [156]. A glaucoma model of retinal ischemia-reperfusion injury in rats showed that oxidative stress initiated vascular senescence, as revealed by the upregulation of SA-β-gal activity, and the senescence-related protein p53, and p16 activity, as well as a reduced RGC viability [157]. The overexpression of cytochrome P450 2J2 inhibited vascular endothelial cell senescence as well as the reduction of pericyte loss by downregulating p53 and p16, which had a protective effect on the survival of RGCs [157]. Besides, in endothelial cells exposed to high glucose or hydrogen peroxide, NADPH oxidase 2 (NOX2) produced excessive ROS, leading to excessive activity of arginase and a decrease in nitric oxide (NO), thereby promoting premature senescence of endothelial cells [158]. Blockade of NOX2 or arginase may inhibit premature senescence by inhibiting the expression of p16 and reducing the release of SASP through an NO dependent pathway. However, further studies are needed to clarify the specific role of vascular endothelial cell senescence in the pathogenesis of glaucoma.

## 4. Cellular senescence as a therapeutic target in glaucoma

There has been an increasing interest in treatments that delay cellular senescence because of the link between aging and glaucoma, with evidence showing that cellular senescence accelerates in glaucoma. In fact, the development of drugs that target specific mechanisms of premature senescence is in fact currently underway. Various medicines and compounds that are currently available, including antioxidants, autophagy inducers, and DNA repair inducers, may prevent premature senescence by altering the mechanisms of action of ROS and oxidative DNA damage. Moreover, epigenetic reprogramming, especially targeting DNA methylation, has been shown to promote tissue repair and the reversal of premature senescence in retina. Furthermore, selective removal of accumulated senescent cells presents a promising therapeutic approach for targeting the senescence phenotype and thereby preventing, delaying, or mitigating glaucoma.

Neurodegenerative diseases, including glaucoma, have been demonstrated to respond well to antioxidant therapy at the cellular and animal levels [159]. As a cofactor in the electron transport chain, coenzyme Q10 plays an essential role in the maintenance of the mitochondrial membrane potential by supporting ATP synthesis, and protecting the mitochondria from free radical damage [160]. Moreover, it promotes RGC survival by maintaining the mtDNA content and the expression of mitochondrial transcription factor A/oxidative phosphorylation complex IV protein in the retina of a mouse model of glaucoma [161].In addition, oral administration of vitamin B3 or gene therapy aimed at boosting the expression of NAD^+^ has been shown to be protective in aged mice for glaucoma prevention and intervention [77]. Metformin is a widely used antidiabetic drug that has been found to target several molecular mechanisms of senescence [162]. Metformin reduced the SASP release caused by retinal ischemia and resulted in a significant decrease in SA-β-gal levels, thus preventing the adverse outcomes of ischemic retinopathy [22]. Although it has been proved to reduce the incidence rate of glaucoma in patients with type 2 diabetes through its anti-inflammatory, antiangiogenic, and antisenescent effects, there is no study showing the effect of metformin on glaucoma in nondiabetic patients [163]. Other antioxidants, such as resveratrol, vitamins D and E, curcumin, ginsenoside, and anthocyanins have also been shown to play a role in the protection against RGC death [164]. Nevertheless, several antioxidants have been reported to fail to exert a neuroprotective effect in clinical trials, possibly as a result of problems with their delivery and their chemical instability [165]. The delivery of antioxidants into the eye via intraocular implants or nanotechnology may be a more effective approach to glaucoma treatment.

Furthermore, mitochondria-targeted antioxidants seem to be more effective than nonspecific antioxidants because they can cross mitochondrial membranes and neutralize ROS at the source [166]. Because some drugs cannot cross the mitochondrial membrane by themselves, they must be conjugated to molecules that are able to enter and localize to the mitochondria, such as triphenylphosphonium (TPP) [167]. The coupling of TPP with coenzyme Q produces MitoQ, a compound that is responsible for supporting healthy aging by reducing aging-related oxidative stress [168]. As shown in animal studies, MitoQ reduces nitrotyrosine (a biomarker of protein oxidation) levels and improves mitochondrial function by increasing the membrane potential of cell organelles [169]. Even though MitoQ has a demonstrated therapeutic potential in mouse models of several neurodegenerative disorders, including Alzheimer's disease, multiple sclerosis and retinopathy, no studies have demonstrated its efficacy in treating glaucoma [170]. SkQ1, which is another TPP-conjugated compound, had a significant therapeutic effect in a rabbit model of glaucoma by reversing the elevation of IOP and several other signs of glaucoma [171]. Nevertheless, a more thorough investigation is required to determine whether antioxidants are effective in preventing glaucoma-associated RGC death by reducing the pro-aging characteristics of the senescent phenotype.

As mentioned above, defective autophagy plays a role in promoting cellular senescence, and mTOR, which is a major protein involved in autophagy and cell growth, has been shown to control SASP by regulating IL-1α and mitogen-activated protein kinase activated protein kinase 2 [172, 173]. As reported recently, the inhibition of mTOR contributes to extending the lifespan and improving mitochondrial function via the attenuation of oxidative stress [85, 174]. Rapamycin-induced activation of REDD1, which is located outside mitochondria and inhibits mTOR signaling, leads to enhanced mitochondrial function and thus in situ rescue of dying RGCs [175]. However, more specific studies need to be conducted to explore the relationships among cellular senescence, autophagy, and neuroprotection strategies in the context of glaucoma.

Increasing evidence indicates that the restoration of epigenetic information and the modification of DNA methylation patterns can lead to the reversal of senescence and the restoration of lesions caused by it [176]. Ectopic expression of *Oct4*, *Sox2* and *Klf4* (OSK) in mouse RGCs was demonstrated to restore the young DNA methylation pattern and transcriptome, thus promoting axonal regeneration following injury and reversing the visual impairment in mouse models with glaucoma and aged mice [177]. To deliver and control OSK expression in mice, they engineered a dual adeno-associated virus (AAV) system under the tight control of a tetracycline response element promoter. This study successfully reversed vision loss after glaucomatous injury has occurred for the first time, which indicated the potential of epigenetic rejuvenation as an antisenescence strategy in glaucoma.

Moreover, the ablation of senescent cells has been proposed as an effective therapeutic approach for targeting the phenotype of cellular senescence and, in turn, help prevent, delay, or mitigate aging-related diseases [178]. Remarkably, despite the removal of only 30% of senescent cells, substantial improvements are still observed in phenotypes associated with aging. A novel class of drugs termed senolytics, which selectively kill senescent cells, have been identified that improve physical function and increase lifespan in old age [179]. A senolytic drug called dasatinib can be used to eliminate endogenous senescent retinal cells by inhibiting the phosphoinositide 3-kinase (PI3K)-AKT pathway after elevation of IOP, thus preventing the loss of retinal function and structure [150]. Quercetin, which is another senolytic drug, has been shown to improve the survival and function of RGCs in glaucomatous neurodegeneration; however, measurements of senescence were lacking [180, 181]. As senolytics have the potential to eradicate senescent cells effectively, these drugs are likely to mitigate the deleterious effects of high IOP on RGC survival in patients suffering from glaucoma and other optic nerve disorders. Although promising evidence has been provided regarding the safety profile of senolytic drugs when used in the visual system, but further research into their potential neuroprotective effects on glaucoma and other neurodegenerative diseases is required [182].

### Conclusions

This review summarizes the knowledge about cellular senescence in glaucoma, and its role in establishing a correlation between aging and glaucoma development. The potential mechanisms of regulation of glaucomatous cellular senescence are currently being elucidated, including oxidative stress, DNA damage, mitochondrial dysfunction, defective autophagy and mitophagy, as is the role of epigenetic reprogramming such as DNA methylation. Accumulated senescent cells, such as TM cells, RGCs, and vascular endothelial cells, may play a role in glaucoma via the secretion of SASP and the elevation of chronic inflammation, thus leading to a loss of RGCs and vision. The development of novel strategies to suppress SASP, eliminate senescent cells, or even reverse senescence seems promising for breakthroughs in glaucoma therapeutics.
